# 24234 Development of a computerized neurocognitive test of interhemispheric transfer for use in pediatric settings

**DOI:** 10.1017/cts.2021.670

**Published:** 2021-03-30

**Authors:** E.I. Pierpont, K. Lim, M. Georgieff, W. Dobyns, M. Luciana, J. Wozniak

**Affiliations:** University of Minnesota

## Abstract

ABSTRACT IMPACT: As newborn screening is now available for X-linked adrenoleukodystrophy, there is a need to establish meaningful disease markers to detect the onset of the severe demyelinating cerebral form of this disease at the earliest possible stage, and to quantify early disease progression to evaluate the relative efficacy of therapies. OBJECTIVES/GOALS: Longitudinal testing of neurocognitive and motor function using smartphone and tablet-based applications holds promise for early detection and quantification of brain white matter changes in patients with adrenoleukodystrophy (ALD) and other rare demyelinating diseases, but this methodology requires validation in pediatric populations. METHODS/STUDY POPULATION: We developed an iPad application with a game-like interface to assess interhemispheric transfer across the corpus callosum, the brain structure where cerebral demyelinating disease typically begins in patients with ALD. Feasibility data from remote test administrations with healthy children were collected to analyze and speed and timing of finger tapping movements requiring bimanual coordination on a touchscreen. RESULTS/ANTICIPATED RESULTS: Among our pilot sample of healthy school-aged children, age-related improvements in finger tapping speed were observed in both single-hand and alternating-hand conditions. Results indicate that remote testing using iPad applications is a viable way to collect psychometric testing data rapidly in pediatric populations and is feasible during a pandemic. Next steps in this research project will be: (1) evaluating the stability of repeated test administrations (test-retest reliability), (2) assessing agreement between performance on our iPad application and validated measures of interhemispheric transfer and fine motor function, and (3) comparing performance of children with known corpus callosum white matter abnormality to performance of healthy children. DISCUSSION/SIGNIFICANCE OF FINDINGS: Brief neurocognitive tests that can be frequently administered may have the ability to capture subtle brain changes in developing children. Approaches enabling remote (virtual) testing will facilitate research during the covid-19 pandemic and are especially well-suited for data collection in rare disease populations.

